# Acute Myocardial Infarction Reduces Respiration in Rat Cardiac Fibers, despite Adipose Tissue Mesenchymal Stromal Cell Transplant

**DOI:** 10.1155/2020/4327965

**Published:** 2020-06-20

**Authors:** Camila I. Irion, Eduarda L. Martins, Michelle L. A. Christie, Cherley B. V. de Andrade, Alan C. N. de Moraes, Raphaela P. Ferreira, Cibele F. Pimentel, Grazielle D. Suhett, Antonio Carlos C. de Carvalho, Rafael S. Lindoso, Adalberto Vieyra, Antonio Galina, Regina C. S. Goldenberg

**Affiliations:** ^1^Carlos Chagas Filho Institute of Biophysics, Federal University of Rio de Janeiro, Rio de Janeiro 21941-902, Brazil; ^2^Leopoldo de Meis Institute of Medical Biochemistry, Federal University of Rio de Janeiro, 21941-902, Brazil; ^3^National Center for Structural Biology and Bioimaging (CENABIO), Federal University of Rio de Janeiro, Rio de Janeiro 21941-902, Brazil; ^4^National Institute of Science and Technology for Regenerative Medicine-REGENERA, Federal University of Rio de Janeiro, Rio de Janeiro, Brazil; ^5^Regenerative Medicine Program, Carlos Chagas Filho Institute of Biophysics, Federal University of Rio de Janeiro, Rio de Janeiro 21941-902, Brazil

## Abstract

Adipose-derived mesenchymal stromal cell (AD-MSC) administration improves cardiac function after acute myocardial infarction (AMI). Although the mechanisms underlying this effect remain to be elucidated, the reversal of the mitochondrial dysfunction may be associated with AMI recovery. Here, we analyzed the alterations in the respiratory capacity of cardiomyocytes in the infarcted zone (IZ) and the border zone (BZ) and evaluated if mitochondrial function improved in cardiomyocytes after AD-MSC transplantation. Female rats were subjected to AMI by permanent left anterior descending coronary (LAD) ligation and were then treated with AD-MSCs or PBS in the border zone (BZ). Cardiac fibers were analyzed 24 hours (necrotic phase) and 8 days (fibrotic phase) after AMI for mitochondrial respiration, citrate synthase (CS) activity, F_0_F_1_-ATPase activity, and transmission electron microscopy (TEM). High-resolution respirometry of permeabilized cardiac fibers showed that AMI reduced numerous mitochondrial respiration parameters in cardiac tissue, including phosphorylating and nonphosphorylating conditions, respiration coupled to ATP synthesis, and maximal respiratory capacity. CS decreased in IZ and BZ at the necrotic phase, whereas it recovered in BZ and continued to drop in IZ over time when compared to Sham. Exogenous cytochrome c doubled respiration at the necrotic phase in IZ. F_0_F_1_-ATPase activity decreased in the BZ and, to more extent, in IZ in both phases. Transmission electron microscopy showed disorganized mitochondrial cristae structure, which was more accentuated in IZ but also important in BZ. All these alterations in mitochondrial respiration were still present in the group treated with AD-MSC. In conclusion, AMI led to mitochondrial dysfunction with oxidative phosphorylation disorders, and AD-MSC improved CS temporarily but was not able to avoid alterations in mitochondria function over time.

## 1. Introduction

Cardiovascular diseases have remained the top major cause of death in the past decades, being responsible for more than 30% of all deaths worldwide, according to the World Health Organization [1]. In 2016, approximately 9.4 million people died of ischemic heart diseases [2], the most common type of cardiovascular disease and a major cause of mortality and morbidity [3]. Myocardial infarction is characterized by sudden ischemic death of myocardial tissue caused by profound ionic and metabolic alterations [4]. The loss of cardiomyocytes is driven by necrotic cell death and activates innate immune pathways resulting in an intense inflammatory response. Subsequently, proliferating fibroblasts undergo myofibroblast differentiation and lead to the formation of a collagen-based scar that maintains structural integrity but loses tissue contractile capacity, characterizing a fibrotic phase after myocardial infarction [5]. At the border zone of myocardial infarcts, cardiomyocytes also undergo metabolic and structural changes as they lose the interaction with the infarcted zone [6]. As a consequence, the border zone presents cardiomyocyte hypertrophy, impaired intercellular conduction, and arrhythmia, representing a significant impact on cardiac function.

Mitochondrial alterations are directly involved in the process of apoptosis and necrosis of cardiomyocytes in the infarcted heart. More than 90% of the energy produced by the heart muscle comes from mitochondrial respiration, i.e., by oxidative phosphorylation [7]. In cardiomyocytes, mitochondria represent approximately 30% of the cell volume and are distributed under the sarcolemma and between myofilaments [7, 8]. Aside from generating ATP, mitochondria are involved in many cellular activities, including ion homeostasis, the generation and removal of reactive oxygen species (ROS), and death regulation [9]. During myocardial infarction, the depletion of oxygen and substrates alters ion homeostasis and results in mitochondrial dysfunction, with alterations in the electron transport system (ETS), decreased ATP production, and reversal of F_0_F_1_-ATP synthase activity (i.e., ATP hydrolysis, rather than production) [10]. These effects impact negatively on the functional recovery of the heart, leading to cardiac dysfunction and loss of cell viability [11, 12].

Given that the cardiac muscle has limited regenerative capacity [13], new methods to treat or prevent myocardium loss following AMI—such as cell therapy—have demonstrated a significant promise. Mesenchymal stromal cells (MSCs) from different niches, including bone marrow [14–15], adipose tissue [16, 17], umbilical cord [18], placenta [19], and menstrual blood [20], have been used to improve cell viability and cardiac function after myocardial infarction.

Different mechanisms have already been extensively proposed to be responsible for the benefits of MSCs such as direct cell replacement [21], paracrine effects [22], secretion of exosomes [23], and immune-modulatory effects [24]. However, additional pathways emerged when i*n vitro* studies showed that MSC can transfer mitochondria to cardiomyocytes [25, 26], and other cell types with mitochondrial dysfunction [27, 28], to restore aerobic respiration and improve cell bioenergetics. *In vivo* studies also showed that bone marrow mesenchymal stromal cells improved cardiac cell metabolism increasing basal glucose uptake and reverted/reduced energetic mitochondrial dysfunction in AMI models, preventing the progression of heart failure and improving contractile performance [29, 30]. Here, we examined changes on mitochondrial respiration (MR) of infarcted cardiac tissue during the necrotic phase (24 h post-AMI) and fibrotic phase (8 days post-AMI) in a rat model of AMI and then studied for the first time the impact of AD-MSC administration in the tissue respiratory capacity.

## 2. Materials and Methods

### 2.1. Animals

Adult female Wistar rats were obtained from the animal house of the Carlos Chagas Filho Biophysics Institute (IBCCF, UFRJ, Rio de Janeiro, Brazil). Female rats were chosen due to their great resistance to myocardial infarction and survival in our hands, thus allowing evaluation of mitochondrial respiration in the late necrotic and fibrotic phases. Animals were given water and food *ad libitum* and were housed at a controlled temperature (23°C), with a 12-hour light-dark cycle. All procedures involving animals were performed according to the Guide for the Care and Use of Laboratory Animals (NIH, USA; 8^th^ edition, 2011) [31], and all protocols involving animals were approved by the committee for the Ethical Use of Animals in Scientific Experimentation from the Federal University of Rio de Janeiro (UFRJ, Brazil; protocol no. 167/13).

### 2.2. Experimental Groups

Forty-eight animals were assigned into the following experimental groups: (i) Sham-operated and nontreated with AD-MSC or PBS (“Sham” group, *n* = 16); (ii) 24 h after AMI induction and PBS treatment (“PBS-24 h” group, *n* = 10); (iii) 8 days after AMI induction and PBS treatment (“PBS-8 d” group, *n* = 7); (iv) 24 h after AMI induction and AD-MSC treatment (“AD-MSC-24 h” group, *n* = 7); and (v) 8 days after AMI induction and AD-MSC treatment (“AD-MSC-8 d” group, *n* = 8).

### 2.3. Cell Isolation and Culture Procedures

Adipose-derived mesenchymal stromal cells (AD-MSC) were obtained from inguinal subcutaneous adipose tissue from male Wistar rats (1.5–2 months old). Briefly, adipose tissue samples were washed with sterile phosphate-buffered saline (PBS), and then, cells were dissociated by enzymatic digestion (0.1% type II Collagenase-Worthington) for 1 hour at 37°C with gentle agitation. After enzyme activity neutralization with Dulbecco's modified Eagle's medium (DMEM) supplemented with 20% fetal bovine serum (FBS), cell suspensions were filtered (BD Falcon™ Cell Strainer 100 *μ*m Nylon) and then spun for 5 min at 300 × g (at room temperature). The supernatant, containing mature adipocytes, was discarded, and the pellet, containing adipose cells, was plated in 100 mm^2^ cell culture flasks, in DMEM-high glucose supplemented with 20% FBS, 2 mM l-glutamine, 100 U/mL penicillin, and 100 mg/mL streptomycin. After 24-hour incubation at 37°C, nonadherent cells were removed by PBS washing, and the cultures were maintained at 37°C in normoxic conditions (21% O_2_ and 5% CO_2_). The medium was changed three times a week, and cells were trypsinized when reaching 80-90% confluence. AD-MSCs at passage 3 were used for cell therapy. The characterization of the cells was previously described by our group [16].

### 2.4. Myocardial Infarction and AD-MSC Transplantation

**Figure 1:**
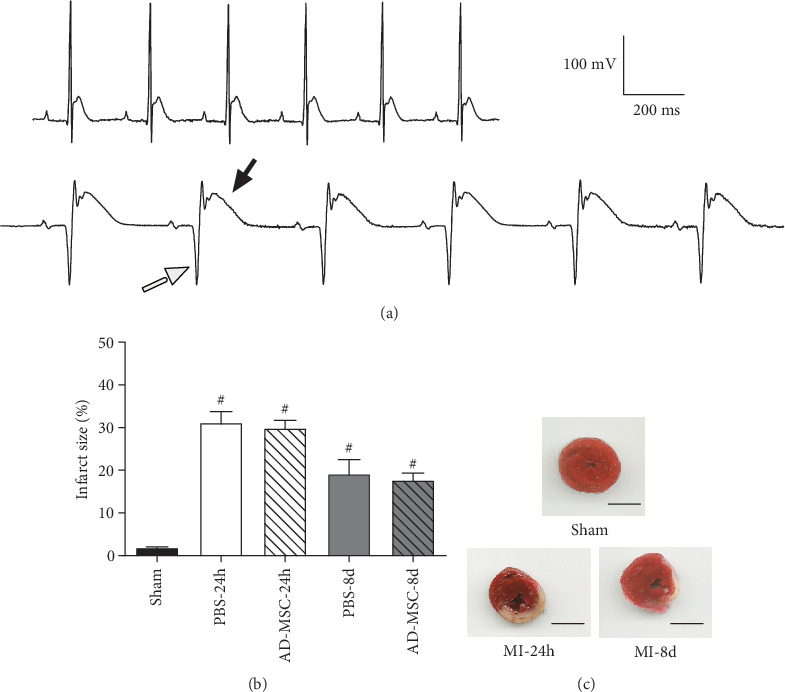
Electrocardiogram (ECG) records and infarct size after acute myocardial infarction (AMI) in female rats. (a) Representative ECG profiles (as analyzed in bipolar lead I) showing no alterations in the Sham group (upper graph), while the ECG of infarcted rats (lower graph) showed a pathological Q wave (empty arrow) and an ST-elevation (filled arrow). Calibration is seen in (a), right side; (b) bar graph showing the infarct size (%) in the PBS treatment and AD-MSC treatment groups after AMI; ^#^*P* < 0.05 in relation to the Sham group. (c) Representative images from the hearts stained with 2,3,5-triphenyltetrazolium from the Sham group and 24 h and 8 d after myocardial infarction. Noninfarcted, viable tissue is stained in red, whereas the infarcted region is unstained (white). Scale bar: 0.4 cm.

Acute myocardial infarction (AMI) was performed in female Wistar rats (200–250 g). Rats were anesthetized with a mixture of ketamine (80 mg/kg) and xylazine (20 mg/kg) by intraperitoneal (IP) route. In the supine position, endotracheal intubation was performed, and rats were mechanically ventilated using an animal respirator (Harvard Apparatus, Holliston, MA) at a rate of 100 breaths/min and stroke volume of 1 to 1.5 mL, depending on the animal weight. AMI was induced by left anterior descending coronary artery (LAD) ligation after heart exposure by thoracotomy. The LAD was permanently occluded with a 6–0 prolene suture (Prolene, Ethicon®, Bridgewater, NY); then the chest was closed, and the rats were allowed to recover for 2 h in a warm mattress and all animals were given postsurgical analgesia (tramadol hydrochloride, 20 mg/Kg). Sham-operated animals (Sham) were subjected to the same procedure, with a suture passed through the heart but without LAD occlusion (*n* = 16). Two hours later, infarcted rats received an intramyocardial injection of 50 *μ*L of 1 × 10^6^ AD-MSC in PBS (treated group) or 50 *μ*L of the vehicle (PBS only) into two sites along the border zone of the left ventricular wall scar tissue. AMI was confirmed by an electrocardiogram (ECG) showing a pathological Q wave and/or an ST-elevation (Figure 1). For infarct size evaluation, the hearts were cut transversally in half and the superior section (part) was incubated in triphenyltetrazolium chloride 1% (TTC) in phosphate buffer (pH 7.4) for 5 minutes at 37°C, fixed in 10% formaldehyde solution, and scanned (Scanjet 2400, HP).

### 2.5. Preparation of Permeabilized Cardiac Fibers

Twenty-four hours (necrotic phase) and 8 days (fibrotic phase) after LAD ligation, animals were euthanized and hearts were excised and placed into ice-cold BIOPS solution [32–34] containing 10 mMCa EGTA buffer (2.8 mM CaK_2_EGTA, 7.2 mM K_2_EGTA), 20 mM imidazole, 50 mM MES (final pH adjusted to 7.1 with KOH at 0°C), 20 mM taurine, 0.5 mM DTT (dithiothreitol), 6.6 mM MgCl_2_ (1 mM free Mg^2+^), 5.8 mM ATP (disodium salt), and 15 mM Na_2_ phosphocreatine, to give 0.1 *μ*M free Ca^2+^ (final concentration) and 160 mM ionic strength. BIOPS was previously frozen in a batch to reduce the influence of batch variability on downstream analyses. The left ventricular fibers from the Sham group and the cardiac fibers from the IZ and BZ from the infarcted rats were manually isolated with the aid of scissors and tweezers and prepared for O_2_ consumption studies according to Pesta and Gnaiger [33] with slight modifications. Briefly, the fibers were permeabilized by gentle agitation for 10 min in 1 mL of ice-cold BIOPS solution containing 25 *μ*g/mL saponin (s7900, Sigma-Aldrich, St. Louis, MO). After permeabilization, fibers were washed twice for 10 min in ice-cold mitochondrial respiration 05 (MIR05) medium containing 110 mM sucrose, 60 mM MES, 20 mM HEPES (final pH adjusted to 7.1 at 0°C by addition of KOH), 0.5 mM EGTA, 0.1% BSA (fatty acid free), 3 mM MgCl_2_, 20 mM taurine, and 10 mM KH_2_PO_4_. After washing, fibers were immediately analyzed in the respirometry system.

The border zone could be differentiated from the ischemic zone by color. The ischemic/fibrotic zone became pale/white color while the border zone is red. The difference between the two zones was clear 24 h and a week after LAD ligation. Red fibers surrounding the pale zone were selected and were considered the border zone. Cardiac muscle biopsies not used for respirometry were store at -70C for further analyses of CS and F_0_F_1_-ATPase activity.

#### 2.5.1. High-Resolution Respirometry

Oxygen consumption rates in permeabilized cardiac fibers were measured at 37°C using an Oxygraph-O_2_K high-resolution respirometry system (Oroboros Instruments, Innsbruck, Austria). Permeabilized fibers (0.8-1.5 mg) were incubated in 2 mL of MIR05 medium. Data recording was performed using the DataLab 4.3.4.70 software (Oroboros Instruments), and respiratory rate was expressed as O_2_ flux per mass [pmol/(s^∗^mg)] and per O_2_ flux per mass [pmol/(s^∗^mg)] corrected by citrate synthase. Adding cytochrome c to evaluate the increase in respiratory rate tested the integrity of the outer mitochondrial membrane; data were eliminated when the increase in the O_2_ consumption was higher than 25% in the Sham group, which was considered a sign of damage to the outer mitochondrial membrane. Experiments were performed in duplicate.

Mitochondrial function was assessed using the multiple substrate-uncoupler-inhibitor titration (SUIT) protocol [33]. Hamilton syringes (Hamilton Company, Reno, NV) were used to add the reagents (above) to the medium to achieve different respiratory states, in the following order: 5 mM pyruvate, 10 mM glutamate, and 2.5 mM malate (PGM), for the nonphosphorylative state (CI); 2.0 mM ADP, for the phosphorylative state associated with complex I activity (CI_p_); 10 *μ*M cytochrome c; 10 mM succinate to activate the complex II via Flavin adenine dinucleotide (FADH2) and to obtain the second phosphorylative state (CI + CII_p_); 2 *μ*g/mL oligomycin (F_0_F_1_-ATP synthase inhibitor) to access the leak state with substrates for CI + CII (*L*); 500-750 nM carbonyl cyanide (p-trifluoromethoxy) phenylhydrazone (FCCP) to achieve the maximal respiratory capacity (*E*); 0.5 *μ*M rotenone, for complex I inhibition (CI_i_); 10 mM malonate, for complex II inhibition (CII_i_); and 2.5 *μ*M antimycin A, for complex III inhibition. Residual oxygen consumption (ROX), which was obtained after inhibition of complexes I, II and III, was used to correct the different mitochondrial respiratory states CI, CI_p_, CI + CII_p_, *L*, and *E* [33]. The O_2_ flux coupled with ATP synthesis (ATPS) was calculated using the following formula: ATPS = CI + CII_p_ − *L*. The complex I contribution to the maximal respiratory capacity (CI_max_) was calculated using the following formula: CI_max_ = *E* − CIi, where CIi corresponds to the O_2_ consumption after the addition of rotenone. The complex II contribution to the maximal respiratory capacity (CII_max_) was calculated using the following formula: CII_max_ = CIi − CIIi, where CIIi corresponds to O_2_ consumption after the addition of malonate.

The following O_2_ flux control ratios (FCR) were also calculated: (i) the leak control ratio that was calculated using the ratio *L*/*E*; (ii) the proportion of the maximal ETS capacity used for ATP production (MEC) that was calculated using the formula MEC = (CI + CII_p_ − *L*)/*E*; (iii) the respiratory control ratio (RCR) that was calculated as RCR = CI + CII_p_/*L*; and (iv) OXPHOS coupling efficiency that was determined by the following equation: 1 − RCR^−1^.

#### 2.5.2. Preparation of Cardiac Muscle Homogenates

Cardiac muscle samples from LV (~50 mg) were homogenized in a lysis buffer containing 50 mM Na_2_HPO_4_/NaH_2_PO_4_ buffer (pH 7.4), 10% glycerol, 1% octyl-phenol ethoxylate, 10 mM Na_3_VO_4_, 10 mM NaF, and 10 mM Na_4_P_2_O_7_ supplemented with the protease inhibitor cocktail P8340 (Sigma-Aldrich), by using a Tissue Ruptor® (Qiagen, Hilden, Germany). After 30 min on ice, tissue lysates were centrifuged at 13,000 × *g* for 20 min at 4°C, and the supernatants were collected and used for citrate synthase and F_o_F_1_-ATPase activity assays, after protein concentration analysis using the Pierce BCA Protein Assay Kit™ (Thermo Fisher Scientific, Waltham, MA).

#### 2.5.3. Citrate Synthase (CS) Activity

CS activity was measured as previously described, with minor modifications [35]. The reaction mixture contained 20 mM Tris-HCl buffer (pH 8.0), 0.42 mM acetyl-CoA, 0.1 mM 5,5′-dithio-bis-(2-nitrobenzoic acid) (DTNB), and 10 *μ*g/mL total protein from the cardiac tissue homogenates. The suspensions were incubated at 37°C for 5 min, and adding 5 mM oxaloacetate started the reaction. The reduction of DTNB by CS was measured at 412 nm in a VICTOR plate reader (Perkin Elmer, Waltham, MA). CS activity, expressed as nmol citrate·mg^−1^·min^−1^, was calculated with the aid of a standard curve of CoA in the presence of DTNB, by using the Origin®8.0 program (OriginLab Corporation, Northampton, MA).

#### 2.5.4. F_0_F_1_-ATPase Activity

To examine whether the respiratory alterations involving the ETS are accompanied by impairment of the catalysis by the molecular machinery responsible for ATP synthesis, we measured the F_0_F_1_-ATPase activity in cardiac muscle fibers after AMI/treatment. The F_0_F_1_-ATPase activity was followed measuring calorimetrically the orthophosphate (P_i_) released from ATP [36]. The reaction mixture (1.25 mL final volume, 37°C) contained 20 mM Hepes-Tris buffer (pH 7.2), 5 mM MgCl_2_, 2 mM ATP, and 0.05 mg/mL protein from cardiac muscle homogenate supernatants. Assays were stopped by adding 12.5 *μ*L of 100% trichloroacetic acid. To estimate the azide-sensitive ATPase activity, the reactions were performed in the absence or presence of 5 mM sodium azide (NaN_3_). Absorbance was read at 600 nm in a VICTOR plate reader (Perkin Elmer), and the results were expressed as nmol P_i_·mg^−1^·min^−1^. The F_0_F_1_-ATPase activity (azide-sensitive) was calculated as the difference between the total ATPase activity in the absence of NaN_3_ and that measured in its presence.

### 2.6. Transmission Electron Microscopy (TEM)

Fragments of LV were fixed in 2.5% glutaraldehyde (diluted in 0.1 M sodium cacodylate buffer, pH 7.2) at 4°C, postfixed for 1 h in 1% OsO4 and 1% K4Fe (CN)6, dehydrated in acetone series (30%, 50%, 70%, 90%, and 100%), and embedded in Poly/Bed® 812 resin (Ted Pella Inc., Redding, CA). Ultrathin sections of embedded tissue were contrasted with uranyl acetate and lead citrate and observed in a JEM-1011 transmission electron microscope (JEOL USA, Peabody, MA). Digital micrographs were captured using an AMT XR80 CCD digital camera (Advanced Microscopy Techniques, Woburn, Massachusetts, EUA) at 12,000x magnification. The total number of mitochondria was quantified using ImageJ (90 images per group). For semiquantitative analysis of mitochondrial abnormalities, electron microscopy images of different areas of myocardial tissue from each group (90 for each) were analyzed according to the following scoring system [37]: grade 0, no or rare mitochondrial alteration; grade 1, limited mitochondrial alteration (loss of cristae and matrix, and cristae disorganization in a few mitochondria); grade 2, multiple or moderate mitochondrial alteration (loss of cristae and matrix material in some mitochondria and crista disorganization in approximately half of the mitochondria); and grade 3, accentuated mitochondrial alteration (cristae fragmentation and loss in most mitochondria and vacuolization). Three blinded observers performed all semiquantitative analyses independently, and the grading data were not subjected to statistical analysis. Other parameters, such as the presence of mitochondrial membrane disruption, the absence of sarcomere, and the presence of collagen were also noted.

### 2.7. Statistics

Comparisons were performed using one-way analysis of variance (ANOVA), followed by Bonferroni's posttest and using an unpaired *t*-test. Data are presented as the means ± standard error (SEM), and *P* values less than 0.05 were considered significant. Statistical analyses were determined using Prism 7.03 software (GraphPad Software Inc., San Diego, CA).

## 3. Results

### 3.1. AMI Leads to Mitochondria Respiration Dysfunction in Permeabilized Cardiac Fibers, Particularly in the Infarcted Zone (IZ)

**Figure 2:**
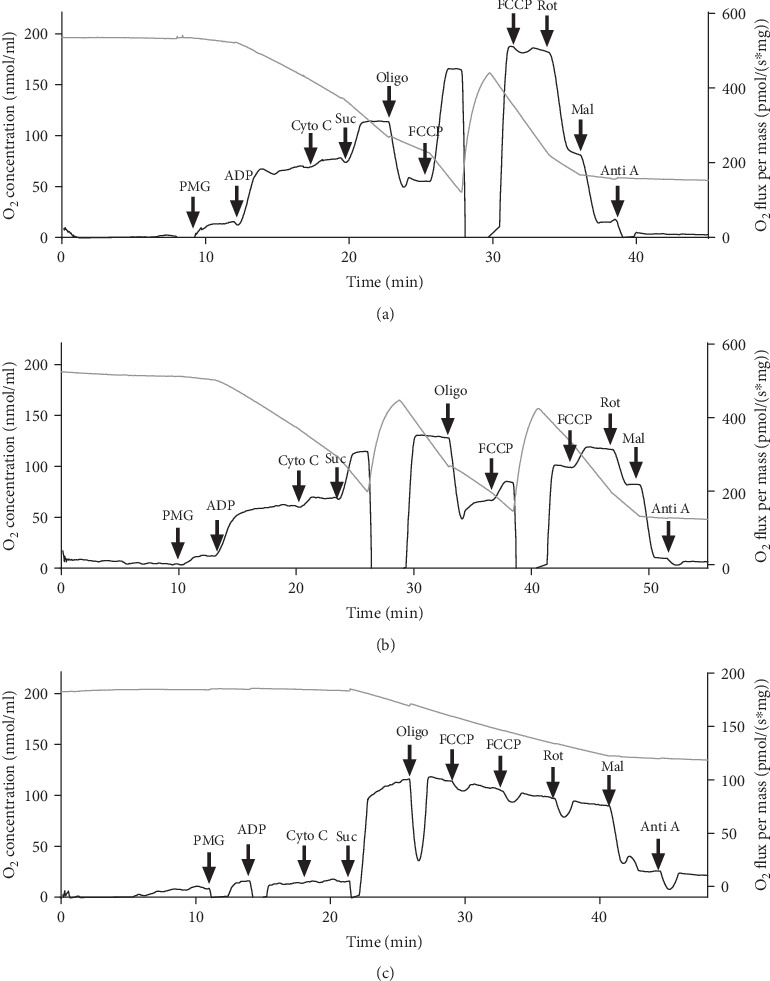
High-resolution respirometry profile of mitochondria in permeabilized cardiac fibers. (a) Representative profile of the O_2_ consumption by using permeabilized cardiac fibers from Sham-operated rats. (b) O_2_ consumption in the border zone (BZ) from rats subjected to AMI; this record was obtained 24 h after AMI, which corresponds to the necrotic phase. (c) O_2_ consumption in the infarcted zone (IZ) from rats subjected to AMI; this record was obtained 24 h after AMI. The O_2_ consumption normalized by tissue mass (black trace) was measured using high-resolution respirometry. Different mitochondrial function parameters were calculated following the multiple substrate-uncoupler-inhibitor titration (SUIT) protocol (arrows), as described in High-Resolution Respirometry. Gray trace represents the O_2_ concentration in the media.

The AMI after LAD ligation was confirmed by ECG analysis, which showed a pathological Q wave and a ST-elevation (Figure 1(a) lower graph), not seen in the Sham-operated rats (Figure 1(a) upper graph). The range of infarct size was between 31.14% ± 2.64 24 h post-AMI and 20.94% ± 3.25 after 8 days (Figures 1(b) and 1(c)). Representative high-resolution respirometry records of saponin-permeabilized fibers from Sham, BZ, and IZ after 24 h are shown in Figures 2(a)–2(c), respectively. The successive mitochondrial respiratory states during the necrotic phase of AMI showed a pronounced global decrease in the responses to substrates, ADP, FCCP, and inhibitors, when the O_2_ fluxes (black traces) from BZ and IZ were compared with those in the Sham group, indicating a decrease in mitochondria activity such as substrate oxidation trough the complexes, ATP production, and electron transfer along the redox centers during noncoupled state in the heart after AMI. PO_2_ (gray traces) represents the O_2_ concentration in the media.

**Figure 3:**
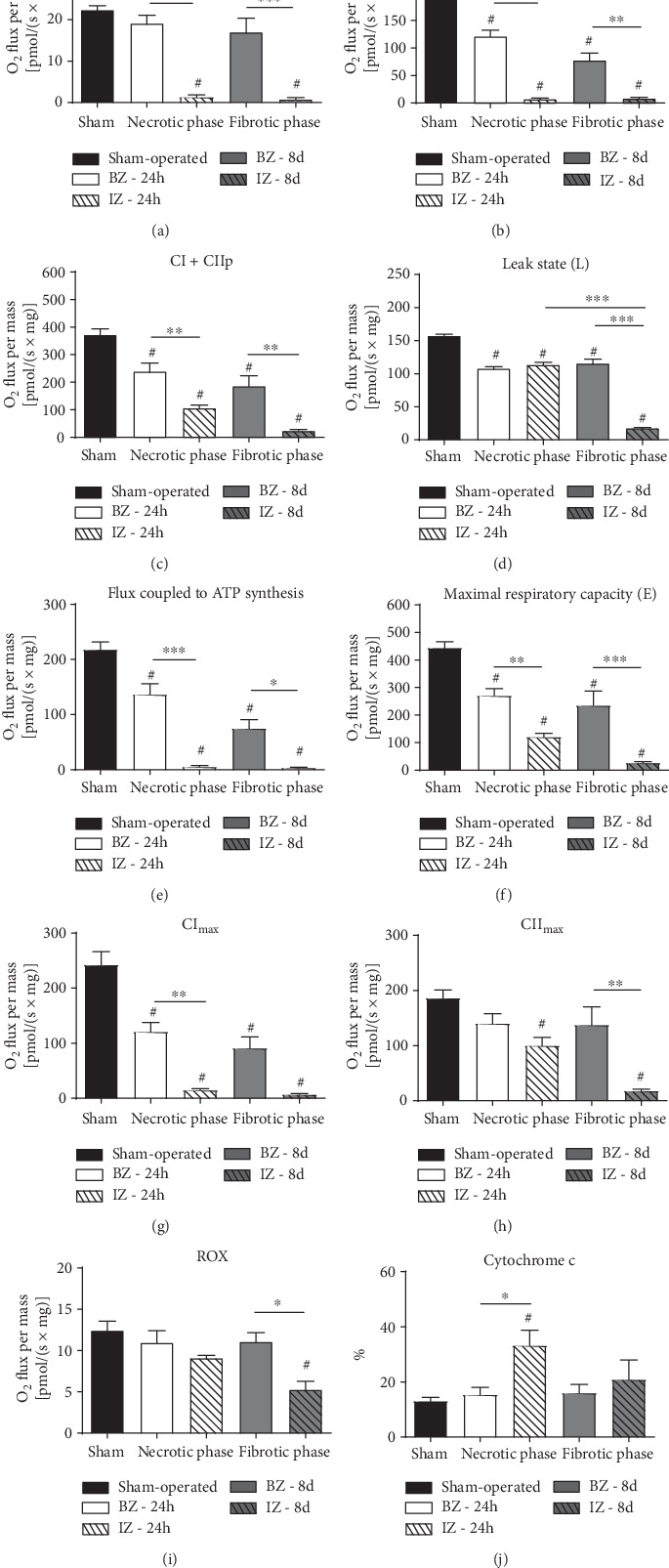
The scrutiny of mitochondrial respiration in the ischemic and border zones, at the necrotic and fibrotic phases, after myocardial infarction (PBS group). The mitochondrial functional parameters described in each panel were obtained or calculated with the use of different substrates in nonphosphorylating and phosphorylating conditions, with specific inhibitors, in the uncoupled state (SUIT protocol) and after addition of cytochrome c, as described in High-Resolution Respirometry. Assays were carried out using permeabilized cardiac fibers. Sham: animals underwent open chest surgery according to the methods. The infarcted zone (IZ) and the border zone (BZ) were analyzed separately. Samples were analyzed 24 h (necrotic phase) or 8 days (fibrotic phase) after AMI induction. Groups are indicated in the abscissa. Respiratory rates are expressed as the O_2_ flux normalized by the muscle wet weight (pmol·s^−1^·mg^−1^) (a–i). Cytochrome c (j) is represented as the percentage. Data represent mean ± SEM values. The number of animals in each group was as follows: Sham group, *n* = 16; PBS-24 h after infarction, *n* = 10; and PBS-8 d after infarction, *n* = 7. ^#^*P* < 0.05 with respect to Sham; when comparisons are made within the 4 BZ and IZ subgroups (dashed lines), the levels of significance are indicated by the number of asterisks: ^∗^*P* < 0.05; ^∗∗^*P* < 0.01; ^∗∗∗^*P* < 0.001 (one-way ANOVA with Bonferroni's posttest).

The quantification of the respiratory damage in mitochondria from fibers isolated from the BZ and IZ during the necrotic (24 h after AMI) and fibrotic (8 days after AMI) phases in the different respiratory states is presented in Figure 3. Mitochondrial respiration at complex I (CI) was severely damaged in the IZ in both phases, when compared to Sham (*P* < 0.001), whereas no alterations were encountered in the BZ (Figure 3(a)). The response to ADP in the presence of glutamate/pyruvate/malate (CI_p_) decreased significantly by 40% and 60% in mitochondria from BZ in the necrotic and fibrotic phases (*P* < 0.001) when compared to the Sham group; the oxygen consumption was barely detectable in mitochondria from IZ in both phases (*P* < 0.001) (Figure 3(b)) indicating a decrease in ATP production after CI stimulation.

In the maximal phosphorylative state (CI + CII_p_) that represents the maximal oxygen consumption after ADP-stimulated supported by glutamate, malate, pyruvate, and succinate, O_2_ consumption after AMI was reduced in all zones and phases (compared to the Sham group, *P* < 0.001). In contrast to CI_p_ oxygen consumption, about 30% of the respiration is still preserved in the IZ at the necrotic phase, but it was minimal in the fibrotic phase.

The leak respiration state (L) (Figure 3(d)), which is the nonphosphorylating resting state when ATP is not active was measured after inclusion of oligomycin and reflects the capacity of the inner mitochondrial membranes to transport H+ by a pathway different from the F0F1-ATP synthase-mediated pathway [38], presenting a significant decrease in both zones during necrotic and fibrotic phases in comparison to the Sham group (*P* < 0.05) and intense alteration in the IZ during the fibrotic phase (*P* < 0.001). When the O_2_ flux coupled to ATP synthesis (CI + CII_p_ − *L*) is examined (Figure 3(e)), we observed the following: (i) it decreased in the 4 AMI subgroups compared to Sham (*P* < 0.001)—the respiration was decreased by more than 30% 24 h after AMI and more than 60% after 8 d AMI in BZ; (ii) it was almost abolished (more than 95%) in mitochondria from the IZ in both the necrotic and fibrotic phases (*P* < 0.001) indicating that cells in this site were not able to produce ATP.

The maximal respiratory capacity or mitochondrial electron transfer capacity along the redox centers during noncoupled state (after FCCP) significantly decreased (*P* < 0.001) in all groups subjected to AMI compared to the Sham group (decreased by 40% in the BZ and 73% in IZ from the PBS-24 h group and 47% in the BZ from the PBS-8 d group), but the most marked reduction occurred again in the IZ in the fibrotic phase (94%, *P* < 0.001) (Figure 3(f)). The complex I contribution to the maximal respiratory capacity (CImax) (Figure 3(g)) decreased in both phases at the IZ (*P* < 0.001), and more than 50% reduction occurred at the BZ (*P* ≤ 0.001). In contrast, the complex II contribution to the maximal respiratory capacity (CII_max_) was not significantly affected by AMI in BZ (besides a reduction of around 25%) but decreased significantly in IZ (*P* = 0.04) in the necrotic phase in relation to the Sham group, being even more inhibited in the fibrotic phase (*P* < 0.001) (Figure 3(h)).

The residual O_2_ consumption (ROX), the nonmitochondrial respiration measured after ETS, was inhibited by the addition of CI, CII, and CIII inhibition, decreased only in the IZ during the fibrotic phase (*P* < 0.001), compared to the Sham group and BZ (*P* < 0.05) (Figure 3(i)). The addition of cytochrome c to the medium demonstrated that the fiber permeabilization process preserved mitochondrial outer membrane integrity, once respiration in the Sham group increased only 13% (Figures 2(a) and 3(j)). A significant increase (33%) was observed in the IZ at the necrotic phase in relation to the Sham group (*P* = 0.0012).

**Figure 4:**
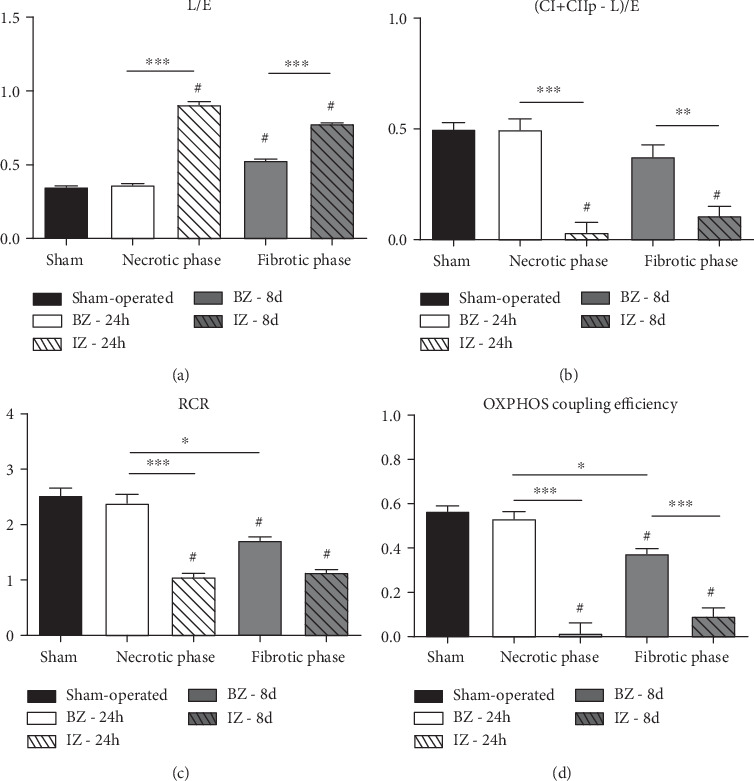
Oxygen flux control ratios (FCR), respiratory control ratio (RCR), and OXPHOS coupling efficiency from the Sham group and rats subjected to AMI (PBS group). Functional parameters were obtained/calculated as described in High-Resolution Respirometry. Data represent the mean ± SEM values. Groups (indicated on the abscissae) and *n* were those described in the legend in Figure 3. ^#^*P* < 0.05 with respect to Sham; for comparisons within the 4 BZ and IZ subgroups, the levels of significance are as follows: ^∗^*P* < 0.05, ^∗∗^*P* < 0.01, and ^∗∗∗^*P* < 0.001 (1-way ANOVA with Bonferroni's posttest).

Internal normalization of the respiratory data by the corresponding maximal respiratory capacity *E* (Figure 4) revealed a significant increase in the leak control ratio (*L*/*E*) in the necrotic and fibrotic phases of IZ when compared with both the Sham group (*P* < 0.001) and BZ group (*P* < 0.001) (Figure 4(a)). In the fibrotic phase, we also observed a modest but significant increase in the leak control ratio in the BZ relative to the Sham group (*P* < 0.05).

The rate of the maximal respiratory capacity used for ATP production (CI + CII_p_ − *L*)/*E* dropped to very low values (ratio < 0.1) only in the IZ (*P* < 0.001) at both phases after AMI—actually, it was barely detectable in the necrotic phase—with no significant changes in the BZ groups (*P* > 0.05) when compared to the Sham group (Figure 4(b)). The mitochondrial efficacy or their capacity to convert its resources into ATP was calculated by RCR and OXPHOS coupling efficiency. The respiratory control ratio (RCR) also decreased in the necrotic phase of IZ (relative to Sham, *P* < 0.001), and this alteration remained at the same level in the fibrotic phase (*P* < 0.001). In BZ the RCR decreased only in the fibrotic phase (*P* = 0.005) (Figure 4(c)). Like RCR, OXPHOS coupling efficiency (Figure 4(d)) was decreased in the necrotic phase of IZ (relative to Sham, *P* < 0.001), and this alteration remained in the fibrotic phase (*P* < 0.001). In BZ, the parameter decreased only in the fibrotic phase (*P* < 0.005) but was significantly better when compared to IZ in the same phase.

### 3.2. AD-MSC Administration after AMI Does Not Preserve the Mitochondrial Function of Permeabilized Cardiac Fibers

With the objective of evaluating if cell therapy could improve and/or prevent the evolution of mitochondrial dysfunction observed after AMI, 1 × 10^6^ AD-MSCs were injected in the BZ of rats 2 hours after AMI. In both phases of AMI (necrotic and fibrotic), AD-MSC administration did not restore mitochondrial respiration after AMI in the border zone, in any of the respiratory states as shown in Table 1. Considering that in this model there was no reperfusion, more damage was observed in the ischemic zone (Table 1S).

### 3.3. Alterations in the Citrate Synthase Activity: AD-MSC Administration after AMI Improves CS Activity during Necrotic Phase

**Figure 5:**
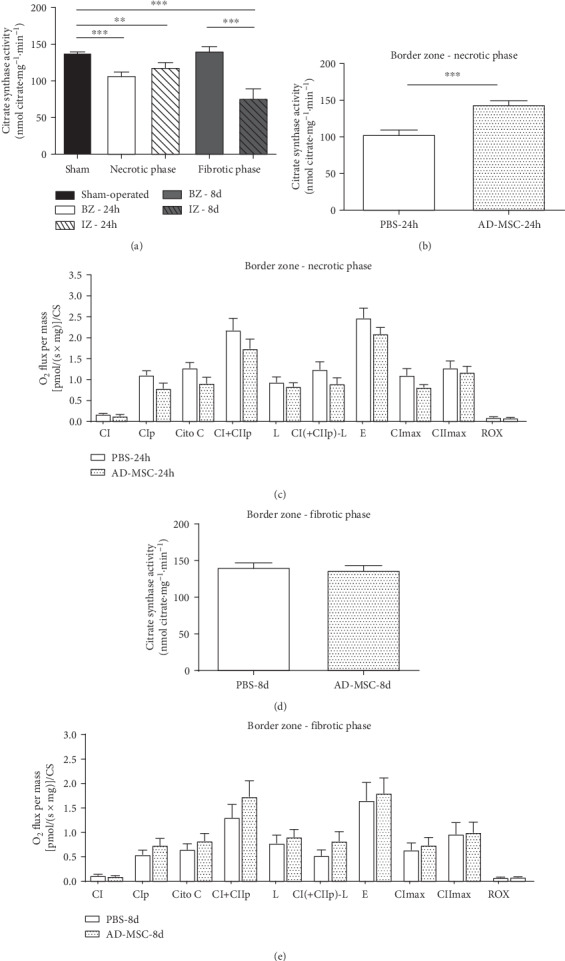
Citrate synthase activity measured in cardiac muscle fibers homogenates and oxygen flux in respirometry experiments normalized to CS activity. The activity is expressed in nmol citrate·mg^−1^·min^−1^ and O_2_ flux by [pmol/(s·mg)]/CS. (a) CS activity from the Sham group and rats subjected to AMI (PBS group). Groups are indicated on the abscissa where white bars represent BZ during the necrotic phase (white) and fibrotic phase (gray) and dashed bars represent IZ during the necrotic phase (white) and fibrotic phase (gray). The number of animals in each group was as follows: Sham group, *n* = 14; PBS-24 h group, *n* = 8; and PBS-8 d group, *n* = 7. (b) CS activity comparing the PBS (*n* = 8) and AD-MSC treatment groups (*n* = 7) in the BZ during the necrotic phase and (d) fibrotic phase (PBS-8 d, *n* = 7; AD-MSC-8 d group, *n* = 8). (c) Mitochondrial functional parameters showing O_2_ flux normalized to CS activity comparing the PBS group and AD-MSC group in the BZ during the necrotic phase and (e) fibrotic phase. *n* of *s*amples were those described in Table 1. Data represent the mean ± SEM values. ^∗∗^*P* < 0.01 and ^∗∗∗^*P* < 0.001 (1-way ANOVA with Bonferroni's posttest). To compare the treated and nontreated groups, an unpaired *t*-test was used (graphs in (b)–(e)).

The citrate synthase (CS) activity is considered an estimation of the mitochondrial content in the tissues [39] and also of mitochondrial integrity [40]. CS activity decreased in both zones in the necrotic phase (*P* < 0.05). In the fibrotic phase, only the CS activity in IZ was significantly lower when compared to Sham (*P* < 0.001), indicating a recovery in the BZ and a continuous decrease in mitochondrial content and integrity in the IZ during AMI progression (Figure 5(a)). Interestingly, when we analyzed samples from the treated group, during the necrotic phase, there was an increase in CS activity in BZ (Figure 5(b), *P* < 0.001) when compared with the PBS-24 h group. In the fibrotic phase, values were similar when comparing the PBS-8 d group and AD-MSC-8 d group (Figure 5(d)). For the IZ, no difference was observed between the PBS and AD-MSC groups (Figure 1Sa and 1Sc).

Because decreases in mitochondrial content generate proportional reductions in the tissue's respiratory capacity [41], results from mitochondrial function parameters were additionally corrected by CS activity, a classical marker of mitochondrial content given the presence of this enzyme inside mitochondrial matrix (Table 2), to evaluate if some of the changes observed in the O_2_ flux after AMI/treatment are due to a decrease in the mitochondrial content of the cardiac tissue. For the BZ, the correction abolished the reduction in some parameters such as CI + CII_p_, *L*, and flux coupled to ATP synthesis observed during the necrotic phase (Figures 3(c)–3(e)), while fibrotic phase alterations were still significant after normalization when compared to the Sham group (*P* < 0.05) with the exception of the *L* parameter. After correction, AD-MSC was not able to improve respiratory capacity (Figures 5(c) and 5(e)) suggesting that the improvement in mitochondria content during the necrotic phase was temporary and it was not enough to avoid alterations in mitochondrial function.

For the IZ, the decrease in the O_2_ consumption observed in numerous mitochondrial functional parameters (Figure 3) remained after normalization (Table 2) in both phases with exception of CII_max_, *L*, and ROX, suggesting that these alterations did not result exclusively from variations in the mitochondrial content in the tissue but reflected genuine changes in mitochondrial function. Treatment with AD-MSC did not affect the O_2_ consumption in this region of the heart (Figure 1S(b) and 1S(d)).

### 3.4. AD-MSC Administration Did Not Promote Recovery of F1F0 ATPase Activity after AMI

**Figure 6:**
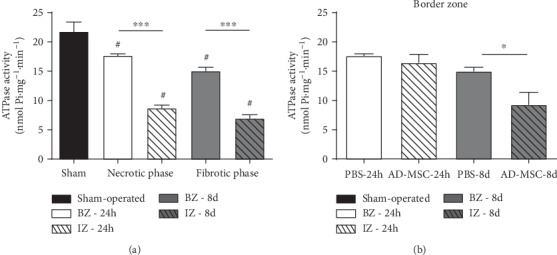
F_0_F_1_-ATPase activity in cardiac muscle homogenates. The activity is expressed in nmol released P_i_·mg^−1^·min^−1^. (a) F_0_F_1_-ATPase activity measured in homogenates of cardiac muscle fibers from the PBS-24 h-treated group (*n* = 4) and PBS-8 d-treated group (*n* = 4) compared with the Sham group (*n* = 11). Groups are indicated on the abscissa where white bars represent BZ during the necrotic phase (white) and fibrotic phase (gray) and dashed bars represent IZ necrotic phase (white) and fibrotic phase (gray). (b) F_0_F_1_-ATPase activity measured in homogenates of cardiac muscle fibers from the PBS-24 h group (*n* = 4) and AD-MSC 24 h group (*n* = 4) in the BZ and from PBS-8 d group (*n* = 4) and AD-MSC-8 d group (*n* = 4). Data represent the mean ± SEM values. (a) ^#^*P* < 0.05 with respect to Sham, within BZ and IZ subgroups (^∗∗∗^*P* < 0.001) (1-way ANOVA with Bonferroni's posttest). (b) *P* < 0.05 (unpaired *t*-test) between the PBS- and AD-MSC-treated groups.

The F0F1-ATPase activity in cardiac muscle fibers after AMI/treatment is presented in Figure 6. For the PBS-24 h group, AMI induced 20 and 60% decreases in the F0F1-ATPase activity in BZ (*P* < 0.001) and IZ (*P* < 0.001), respectively, when compared to the Sham group. Enzyme activity was also lower in the fibrotic phase (PBS-8 d group), in both BZ and IZ, 33 and 70% (*P* < 0.001) less when compared to the Sham group. In both phases, F0F1-ATPase activity was significantly lower in the IZ when compared to the BZ (*P* < 0.001; Figure 6(a)). Treatment with AD-MSCs was not able to revert the reduction in the ATPase activity in the BZ (Figure 6(b)) and in the IZ (data not shown).

**Figure 7:**
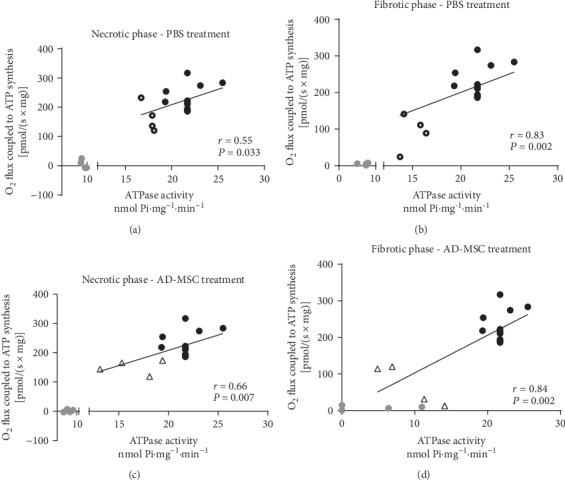
Linear correlation between F_0_F_1_-ATPase activity and O_2_ flux coupled to ATP synthesis. (a, c) Necrotic phase. (b, d) Fibrotic phase. Points corresponding to Sham (black circles), IZ (gray circles), and BZ (empty circles and triangle) were obtained from the F_0_F_1_-ATPase data presented in Figure 6 and the paired data of O_2_ consumption coupled to ATP synthesis. Correlations were analyzed only with the use of Sham and BZ data. The IZ values, included for the illustration in the figure, were not used in the correlations because O_2_ consumption coupled to ATP synthesis did not differ from zero. The *r* and *P* values calculated by the least square method as indicated within each panel.

Then, we evaluated whether the F0F1-ATPase activity correlates with the O_2_ flux coupled to ATP synthesis, in Sham and in BZ zone (PBS and AD-MSC groups) at the 2 phases after AMI (Figure 7). The data from IZ were not included in the statistical analysis because O_2_ coupled to ATP synthesis was close to zero (Figure 3(f)). Linear regression analysis revealed a significant correlation between F0F1-ATPase activity and the O_2_ flux coupled to ATP synthesis in a wide range of values for both parameters in Sham and BZ in both the necrotic (Figure 7(a), *r* = 0.55 and *P* = 0.033) and fibrotic phases (Figure 7(b), *r* = 0.83 and *P* = 0.002). AD-MSC treatment did not result in benefits (Figures 7(c) and 7(d)). These data suggest that the reductions of O_2_ coupled to ATP synthesis are due to damage in the F0F1-ATPase moiety and consequently impaired catalytic activity in the forward mode of synthesis.

### 3.5. AMI Results in Ultrastructural Alterations in Cardiac Tissue Mitochondria despite Treatment with AD-MSC

**Figure 8:**
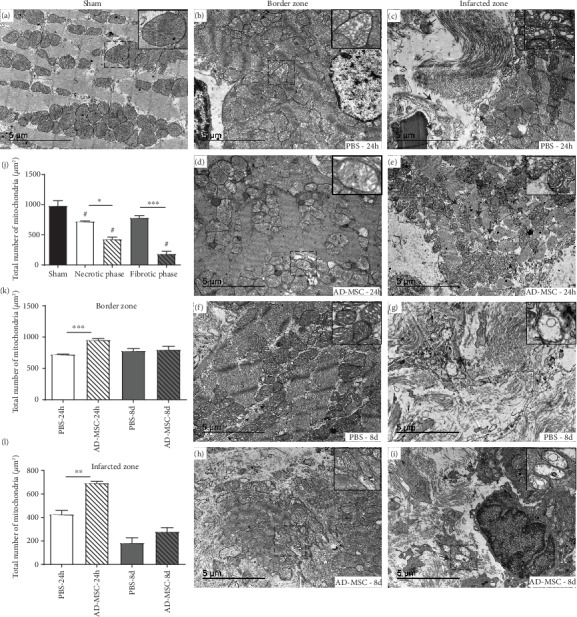
Representative images of transmission electron microscopy analysis of cardiac tissue at the necrotic and fibrotic phase after AMI. (a) Sham-operated group, where the inset presents typical mitochondria (M) with well-structured cristae and dense matrix. Image of (b) BZ and (c) IZ from the PBS-24 h-treated group, which displayed mitochondrial membrane disruption (black arrow); the inset allows the visualization of small and distorted cristae together with matrix vacuolization. Collagen deposits indicative of fibrosis (F) are also visible in the IZ. (d) AD-MSC-24 h group showing a moderate amount of mitochondria between sarcomeres (S); the inset allows the visualization of slight disturbance of mitochondrial architecture. (e) IZ of AD-MSC-treated rats, showing mitochondrial membrane disruption, with multiple alterations in the mitochondria, including loss of cristae and matrix material, and crista disorganization in approximately half of all mitochondrial profiles. From fibrotic phase (f), BZ from rats treated with PBS shows multiple and variable ultrastructural alterations, including disorganization of the mitochondrial cristae, and loss of cristae, and matrix material in some mitochondria. (g) IZ from PBS-treated shows significant and accentuated ultrastructural alterations such as mitochondrial membrane disruption (black arrow) and fewer number of mitochondria, which had reduced matrix density and fewer or no cristae, as well as internal vacuoles. The cardiac tissue displayed the areas of fibrosis (F) with no myofibrils. Images from the fibrotic phase in the (h) BZ and (i) IZ from AD-MSC-treated rats show the same aspects observed in the PBS-treated conditions; (j) the total number of mitochondria from the PBS-24 h-treated group (*n* = 3) and PBS-8 d-treated group (*n* = 3) compared with the Sham group (*n* = 3); (k) the total number of mitochondria from BZ comparing the PBS group (*n* = 3) and AD-MSC group (*n* = 3) in the necrotic and fibrotic phase; and (l) the total number of mitochondria from IZ comparing the PBS group (*n* = 3) and AD-MSC group (*n* = 3) in the necrotic and fibrotic phase. Data represent the mean ± SEM values. (j) ^#^*P* < 0.05 with respect to Sham; within the BZ and IZ subgroups, the levels of significance are indicated by the number of asterisks: ^∗^*P* < 0.05, ^∗∗^*P* < 0.01, and ^∗∗∗^*P* < 0.001 (1-way ANOVA with Bonferroni's posttest). To compare the treated and nontreated groups, an unpaired *t*-test was used (k, l). Other symbols in the panels—M: mitochondria; N: nucleus; S: sarcomeres. Scale bar = 5 *μ*m.

TEM revealed visible alterations in the mitochondria in the groups that had been subjected to AMI (Figure 8). In the Sham group (Figure 8(a)), we observed well-organized and densely packed cardiac myofibrils forming sarcomeres and a great number of mitochondria (982.3 ± 84.04 mitochondria/*μ*m^2^) located between the myofibrils (Figure 8(j)). In this group, mitochondria appeared intact, with a well-defined double membrane surrounding the mitochondrial matrix, abundant cristae, and a dense matrix (grade 0). The mitochondrial shape varied between tubular and spherical.

In infarcted groups, TEM analysis revealed different degrees of mitochondrial damage compared with the Sham group (grades 1-3) associated with a decreased in the mitochondrial number (Figure 8j). The mitochondrial number decreased in both zones in the necrotic phase (BZ = 721.3 ± 9.24, IZ = 425.3 ± 35.3; *P* < 0.05). In the fibrotic phase, only IZ (182.7 ± 44.03 mitochondria/*μ*m^2^) presented a significantly decreased number when compared to Sham (*P* < 0.001).

When comparing the treated and nontreated groups, at the necrotic phase in the BZ, in both the PBS-24 h group and AD-MSC-24 h group (Figures 8(b) and 8(d)), we observed that the majority of mitochondria presented an intact double membrane, with rare or few mitochondria showing membrane disruption, and some mitochondria displaying disorganized and distorted cristae (grade 1). Interestingly, the AD-MSC group presented a significant increase in the number of mitochondria (953.7 ± 24.32 mitochondria/*μ*m^2^) when compared to the PBS-24 h group (721.3 ± 9.24 mitochondria/*μ*m^2^; Figure 8(k)). At the same phase, the IZ (PBS-24 h group) (Figure 8(c)) also showed the presence of mitochondrial membrane disruption and alterations compatible with grade 1. In the IZ, the AD-MSC-24 h group presented alterations compatible with grade 2, besides having a significantly greater amount of mitochondria (693 ± 16.26 mitochondria/*μ*m^2^) in relation to the PBS-24 h group (425.3 ± 35.3 mitochondria/*μ*m^2^) (Figure 8(l)).

In the fibrotic phase, the BZ from the PBS-treated group had a great number of mitochondria (780.7 ± 37.95 mitochondria/*μ*m^2^) between the sarcomeres and variable or no mitochondrial abnormalities (grades 0-3), depending on the animal (Figure 8(f)). BZ from the AD-MSC-8 d group displayed alterations compatible with grade 2, with no mitochondrial membrane disruption (Figure 8(h)) and no difference in mitochondria content (798.3 ± 56.1 mitochondria/*μ*m^2^) when compared to the PBS-treated group (Figure 8(k)). Finally, in the IZ, no visible difference was observed between the PBS-8 d group (most part of the samples in grade 3) and AD-MSC-8 d group (grades 2-3). Both groups (Figures 8(g) and 8(i)) showed the areas of fibrosis and lacked intact myofibrillar structure. Mitochondria displayed membrane rupture, loss of cristae/matrix material, and internal vacuoles. No difference was observed in the number of mitochondria between groups (Figure 8(i)).

## 4. Discussion

Mitochondrial dysfunction is a key event involved in cardiac ischemic lesions, which represent the most common fatal events. The present work shows alterations in the respiratory capacity of cardiac tissue after AMI. In addition, we show for the first time that AD-MSC transplantation in this model does not prevent the development of mitochondrial dysfunction but promotes an increase in the number of mitochondria and transient improvement of CS activity in an early period after AMI.

We initially investigated the correlation between respiratory parameters in different functional states, especially those associated with ATP synthesis. Our data are in general very comparable with the literature [42, 43] with respect to the respiratory parameters of oxygen flux (expressed in wet weight (ww)). Similar to previous reports [29, 44, 45], we also showed that AMI decreased mitochondrial respiration in cardiac cells in different functional states, mainly those associated to ATP synthesis, with ultrastructural modifications in an acute lesion scenario. The feeding by electrons of the ETS in the necrotic phase was more affected through complex I (NADH: ubiquinone oxidoreductase) than that starting from the succinate dehydrogenase (complex II), although both were completely damaged in the fibrotic phase of the IZ (IZ still responded to succinate addition during the necrotic phase). The difference could be ascribed to the higher number of subunits of complex I when compared to complex II [46, 47], which could confer less stability to the former facing increased mitochondrial Ca^2+^ and an elevated production of O_2_ and N2 reactive species (ROS and NRS, respectively) [48, 49]. Also, it is known that complex I is more vulnerable as described in other ischemic models like cold ischemia in pigs [44] and in cold ischemia followed by reperfusion in rats [50]. In the alterations in the BZ, both the significant decline of respiration in the states CI_p_ andCI + CII_p_and the overall O_2_ flux coupled to ATP synthesis demonstrate that the biochemical impairment of cardiac cells, which occurs beyond the ischemic area, and its extent could be important in determining the fate of heart function recovery after infarction.

In the IZ, the absent response to ADP addition in both phases, associated with values close to zero in the flux coupled to ATP synthesis (which encompasses all reactions involved in the ADP phosphorylation to form ATP) and the reduction in the O_2_ consumption in these same states in the BZ, could be explained by the blockage or reversal of complex V activity. Instead of producing ATP, the enzyme could be hydrolysing ATP (generated by anaerobic glycolysis) to restore the electrochemical gradient that is lost under ischemic conditions [51, 52]. In our work, the complex V dysfunction was confirmed by FoF1-ATPase activity analysis. The profile depicted in Figure 6 indicates a reduction the enzyme activity in the BZ in both phases and an accentuated initial impairment found in the IZ persists in the fibrotic phase. These observations are indicative of structural and functional disarrangement of the different subunits of the complex, which result from the alterations encountered in the cristae, a hypothesis supported by the evidence of a mutual relationship between cristae integrity and structural stability of the quaternary organization of F0F1-ATPase and of its dimerization [53, 54]. Also, the damage in complex V forward mode (ATP synthesis) in the BZ is demonstrated by the positive correlation found when O_2_ consumption coupled to ATP synthesis is correlated with F0F1-ATPase activity (Figure 7). This correlation does not apply in the IZ, when high rates of hydrolysis are encountered and O_2_ flux coupled to ATP synthesis is not different from zero (Figure 3(f)). Possibly, the F0F1-ATP synthase moiety is not totally destructured, whereas the electron flux is mainly at the level of complex I. The preservation of the sensitivity to oligomycin (Figure 3(e)) has been considered a proof against the complete destructuration of the F0F1-ATP synthase [55]. This view is supported after the analysis of the velocity of electron flux along the complexes toward the final step at complex IV when the respiration is uncoupled from ATP synthesis; i.e., when the transmembrane gradient potential is dissipated by the addition of FCCP, revealing that the electron transfer itself along the different Fe–S redox centers is altered in AMI in a comparable extent with the ATP synthesis capacity. The identical profiles of O_2_ consumption in CI + CII_p_ and *E* states (Figures 3(c) and 3(g)) allow the demonstration of the impairment of electron transfer along with the redox centers, mainly at the level of complex I.

The disturbances in the mitochondrial architecture seen in Figures 8(b)–8(i) after AMI, with the gradation of lesions encountered when BZ and IZ are compared, certainly affect the efficiency of H+ gradient utilization, which is partially dissipated as heat through the leakage pathway [12]. The increase in the L/E ratio of respiration (Figure 4(a)) mainly in the IZ group (with values close to 1), together with the decrease in the (CI + CII_p_ − *L*)/*E* (Figure 4(b)), two of the FCR investigated in the present study, reveals that the H+-driving force established across the mitochondrial internal membrane as the result of the electron fluxes is being lost mainly via H+ leakage (more than 90%) and is not being used to produce ATP via the F0F1-ATP synthase after AMI. These functional alterations were evident in the IZ, with no modifications in BZ—except for L/E at the fibrotic phase—thus probably contributing to the total collapse of ATP synthesis in IZ and to the partial collapse of ATP synthesis in BZ (Figure 3(f)). The fact that the electron flux along with the redox centers within the complexes—mainly at the level of complex I—is also compromised, aggravates the effect of the ischemia-induced H+ leak. Finally, the accentuated increase in respiration after addition of cytochrome c (Figure 3(k)) at the acute phase in IZ—which reveals important damage in the internal mitochondrial membrane and cytochrome c release—could be considered a key and transient event determining the fate of the fibers towards cardiomyocyte death. The increase that doubles the accepted normal values around 15% [56] shows the trigger of the apoptotic cascade [45, 57, 58], which progressively culminates with late left ventricle dysfunction. The normalized values of cytochrome c found in IZ during fibrotic phase could be justified by the low response to most part of the reagents (Figure 3).

The functional and permanent alterations in O_2_ flux in the different functional states are not totally dependent on the decreased number of mitochondria and/or in the mitochondrial integrity once CS activity returned to Sham values in the BZ in the fibrotic phase. The correction of the O_2_ flow for the most part of the parameters by CS activity (Table 2) also confirms that the respiratory dysfunction is still present independent of the number of mitochondria.

After understanding the mechanisms involved in mitochondria dysfunctions promoted by AMI, we aimed to determine if the protective effects of AD-MSC were also associated with mitochondria protection. MSC are considered a promising cell resource for regenerative medicine, and different groups showed the benefits of MSC transplantation into the heart function after AMI [16, 17, 59]. However, only a few of them analyzed mitochondrial dysfunction and bioenergetics using MSC and there is no consensus on the mechanisms related to the benefits achieved with cell therapy [29, 30, 60, 61]. Therefore, we decided to use AD-MSC transplantation in the AMI model.

In mice resistant to insulin, Hughey et al. [29] reported that BM-MSC preserved the peri-infarct insulin-stimulated fatty acid uptake (remote zone) and prevented increases in glucose uptake in the peri-infarcted region after AMI. With high-resolution respirometry, they also showed that BM-MSC decreased the O_2_ consumption associated with the nonphosphorylative state and did not improve the CI_p_, the CI + CII_p_, and CS activity; however, the cells were able to prevent the decrease in the RCR after AMI, suggesting that BM-MSC preserved the mitochondrial coupling efficiency and the response to ATP. On the other hand, Eun et al. [61] reported that BM-MSC transplantation into the cardiac tissue of rats after permanent AMI did not restore the levels of glycolysis and ATP synthesis-related metabolites, which were decreased 7 days post-AMI. In our work, using AD-MSC, we showed that cells were able to increase CS activity and total mitochondrial number in the necrotic phase of AMI in the border zone when compared to the nontreated group, but this was not enough to avoid alterations in mitochondrial function, including the phosphorylative and nonphosphorylative states, F_0_F_1_-ATPase activity, and parameters like RCR and OXPHOS coupling efficiency. RCR is a good indicator of mitochondrial dysfunction once changes in OXPHOS parameters can modify the RCR [38]. A low RCR, after AMI, as shown in our results in the BZ and IZ (in PBS and AD-MSC treated groups), can indicate that mitochondria have a low capacity for substrate oxidation and ATP turnover and/or high proton leakage. The antiapoptotic effect of AD-MSC in the *in vivo* and *in vitro* studies [22, 62] plus the transfer of mitochondria [25, 26] could be a good explanation why in this work we found a temporary improvement in CS activity, a way to estimate mitochondrial content. Nevertheless, such possibility does not justify why this increase was not followed by improvement of the mitochondrial function (Table 1) or even resulted in a reduction in the scar area in the MI hearts (Figure 1(c)). Also, TEM analysis from infarcted animals confirmed that functional changes in mitochondria were followed by morphologic alterations and were still present in treated groups (Figure 8). Finally, although AD-MSC promoted some beneficial effects by increasing the mitochondria number after AMI and improving CS activity in the necrotic phase, it was not sufficient to overcome the damages promoted by AMI.

## 5. Conclusions

In conclusion, the dissection of the complexes of electron fluxes through the mitochondrial complexes, in a scenario of crista destructuring, revealed that AMI preferentially and irreversibly affected the NAD^+^- rather than the FAD-dependent oxidoreduction reactions in cardiac mitochondria. Moreover, the strongly depressed respiration coupled to ATP synthesis in BZ demonstrated the impairment of phosphorylation extending beyond the ischemic area. The damage in F_0_F_1_-ATP synthase and the loss of complex I function in both zones, together with an initial burst of cytochrome c release at the acute phase in IZ, likely initiate progressive biochemical and structural cardiac remodeling also in the periphery of the initially ischemic area. The treatment with AD-MSCs did not prevent or improve OXPHOS alterations generated by AMI but was able to transiently prevent the decrease of CS activity.

## Figures and Tables

**Table 1:** Effects of mesenchymal stromal cells from adipose tissue (AD-MSCs) on the mitochondrial function of permeabilized cardiac fibers (BZ) from rats after acute myocardial infarction (AMI). Rats were treated with AD-MSCs or PBS after AMI, and different parameters of the mitochondrial function of permeabilized cardiac fibers were analyzed by high-resolution respirometry, according to the multiple substrate-uncoupler-inhibitor titration protocol.

Mitochondrial function parameter (in pmol O_2_ s^−1^ mg^−1^)	Necrotic phase		Fibrotic phase	
	PBS (*n* = 10)	AD-MSC (*n* = 7)	PBS (*n* = 7)	AD-MSC (*n* = 8)
CI	19.12 ± 1.95	19.14 ± 4.93	16.97 ± 3, 42	13.33 ± 2.16
CI_p_	120.98 ± 11.40	113.0 ± 17.90	77.65 ± 12.86	97.79 ± 18.31
CI + CII_p_	238.72 ± 30.61	249.02 ± 31.95	185.57 ± 37.49	229.50 ± 42.83
Leak state (*L*)	102.5 ± 13.61	119.9 ± 12.61	110.86 ± 22.99	120.18 ± 20.10
(CI + CII_p_ − *L*)	136.19 ± 19.59	129.11 ± 20.1	74.71 ± 16.19	109.32 ± 25.21
*E*	270.24 ± 25.41	299.31 ± 22.06	234.79 ± 52.24	238.99 ± 40.95
CI_max_	120.52 ± 17.17	117.38 ± 8.89	91.15 ± 20.35	98.10 ± 20.85
CII_max_	139.98 ± 17.82	168.23 ± 19.91	137.13 ± 33.29	132.28 ± 27.80
ROX	10.93 ± 1.48	12.18 ± 1.87	11.06 ± 1.10	11.28 ± 0.94
Cyto C (%)	15.30 ± 2.77	14.50 ± 1.8	15.90 ± 3.22	13.0 ± 2.76
L/E	0.37 ± 0.003	0.39 ± 0.01	0.509 ± 0.039	0.512 ± 0.04
(CI + CII_p_–*L*)/*E*	0.50 ± 0.05	0.42 ± 0.05	0.37 ± 0.05	0.42 ± 0.05
RCR	2.39 ± 0.16	2.04 ± 0.11	1.72 ± 0.06	1.85 ± 0.14
OXPHOS coupling efficiency	0.53 ± 0.03	0.47 ± 0.04	0.37 ± 0.02	0.41 ± 0.05

CI: nonphosphorylative state; CI_p_: phosphorylative state associated with complex I; CI + CII_p_: maximal phosphorylative state; (CI + CII_p_ − *L*): flux coupled with ATP synthesis; *E*: maximal respiratory capacity; CI_max_: complex I contribution to the maximal ETS capacity; CII_max_: complex II contribution to the maximal ETS capacity; ROX: residual oxygen consumption; Cyto C: an index of mitochondrial outer membrane integrity; *L*/*E*: leak control ratio; (CI + CII_p_ − *L*)/*E*: expressing how much from ETS capacity is used to produce ATP; RCR: respiratory control ratio. Values are expressed as the mean ± SEM. Any difference was visible between the vehicle-treated (PBS) group and the AD-MSC-treated group (by unpaired *t*-test).

**Table 2:** Oxygen flux in respiratory experiments normalized to CS activity. Effects of AMI on the mitochondrial function of permeabilized cardiac fibers from rats after acute myocardial infarction (AMI) during the necrotic and fibrotic phases. Different parameters of the mitochondrial function of permeabilized cardiac fibers were analyzed by high-resolution respirometry, according to the multiple substrate-uncoupler-inhibitor titration protocol.

Mitochondrial function parameter (in pmol O_2_ s^−1^ mg^−1^/CS)		Necrotic phase		Fibrotic phase	
	Sham group (*n* = 16)	BZ (*n* = 10)	IZ (*n* = 10)	BZ (*n* = 7)	IZ (*n* = 7)
CI	0.16 ± 0.008	0.18 ± 0.018	0.011 ± 0.005^#,^^∗∗∗^	0.12 ± 0.024	0.009 ± 0.005^#,^^∗∗∗^
CI_p_	1.39 ± 0.078	1.11 ± 0.104^#^	0.06 ± 0.02^#,^^∗∗∗^	0.55 ± 0.091^#^	0.10 ± 0.026^#,^^∗^
CI + CII_p_	2.7 ± 0.15	2.19 ± 0.28	0.95 ± 0.095^#,^^∗∗∗^	1.31 ± 0.26^#^	0.29 ± 0.06^#,^^∗∗^
Leak state (*L*)	1.12 ± 0.09	0.94 ± 0.125	0.97 ± 0.13	0.78 ± 0.16	0.25 ± 0.05^#,^^∗^
(CI + CII_p_ − L)	1.58 ± 0.10	1.25 ± 0.18	0.05 ± 0.022^#,^^∗∗∗^	0.53 ± 0.11^#^	0.04 ± 0.013^#,∗^
E	3.22 ± 0.16	2.48 ± 0.23^#^	1.08 ± 0.13^#,^^∗∗∗^	1.66 ± 0.37^#^	0.32 ± 0.06^#,^^∗∗^
CI_max_	1.76 ± 0.17	1.10 ± 0.16^#^	0.13 ± 0.024^#,^^∗∗∗^	0.64 ± 0.14^#^	0.08 ± 0.022^#^
CII_max_	1.35 ± 0.11	1.28 ± 0.16	0.89 ± 0.14	0.97 ± 0.24	0.22 ± 0.04^#,^^∗∗^
ROX	0.09 ± 0.008	0.10 ± 0.013	0.08 ± 0.003	0.078 ± 0.008	0.064 ± 0.012
Cytocrome C (%)	13.35 ± 1.43	14.53 ± 3	86.68 ± 26.7^#,^^∗∗^	15.66 ± 3.03	30.59 ± 11.58

CI: nonphosphorylative state; CI_p_: phosphorylative state associated with complex I; CI + CII_p_: maximal phosphorylative state; *L*: leak state; (CI + CII_p_ − *L*): flux coupled with ATP synthesis; *E*: maximal respiratory capacity; CI_max_: complex I contribution to the maximal ETS capacity; CII_max_: complex II contribution to the maximal ETS capacity; ROX: residual oxygen consumption; Cyto C: an index of mitochondrial outer membrane integrity (represented as percentage). Data represent the mean ± SEM values. ^#^*P* < 0.05 with respect to Sham; when comparisons are made within the 4 BZ and IZ subgroups (horizontal lines), the levels of significance are indicated by the number of asterisks: ^∗^*P* < 0.05; ^∗∗^*P* < 0.01; ^∗∗∗^*P* < 0.001 (1-way ANOVA with Bonferroni's posttest).

## Data Availability

The data used to support the findings of this study are included within the article.

## Supplementary Materials

Supplementary Materials Figure 1S: citrate synthase activity measured in cardiac muscle fiber homogenates and oxygen flux in respirometry experiments normalized to CS activity in the IZ. Table 1S: effects of mesenchymal stromal cells from adipose tissue (AD-MSCs) on the mitochondrial function of permeabilized cardiac fibers (IZ) from rats after acute myocardial infarction (AMI).
