# Supplementary material for: Acute Myocardial Infarction Reduces Respiration in Rat Cardiac Fibers, despite Adipose Tissue Mesenchymal Stromal Cell Transplant
Source: Stem Cells Int. 2020 Jun 20;2020:4327965. doi: 10.1155/2020/4327965 (PMC7322589; doi:10.1155/2020/4327965)
Supplement: Supplementary Materials — Figure 1S: citrate synthase activity measured in cardiac muscle fiber homogenates and oxygen flux in respirometry experiments normalized to CS activity in the IZ. Table 1S: effects of mesenchymal stromal cells from adipose tissue (AD-MSCs) on the mitochondrial function of permeabilized cardiac fibers (IZ) from rats after acute myocardial infarction (AMI). [file 4327965.f1.pdf]

Supplementary Materials

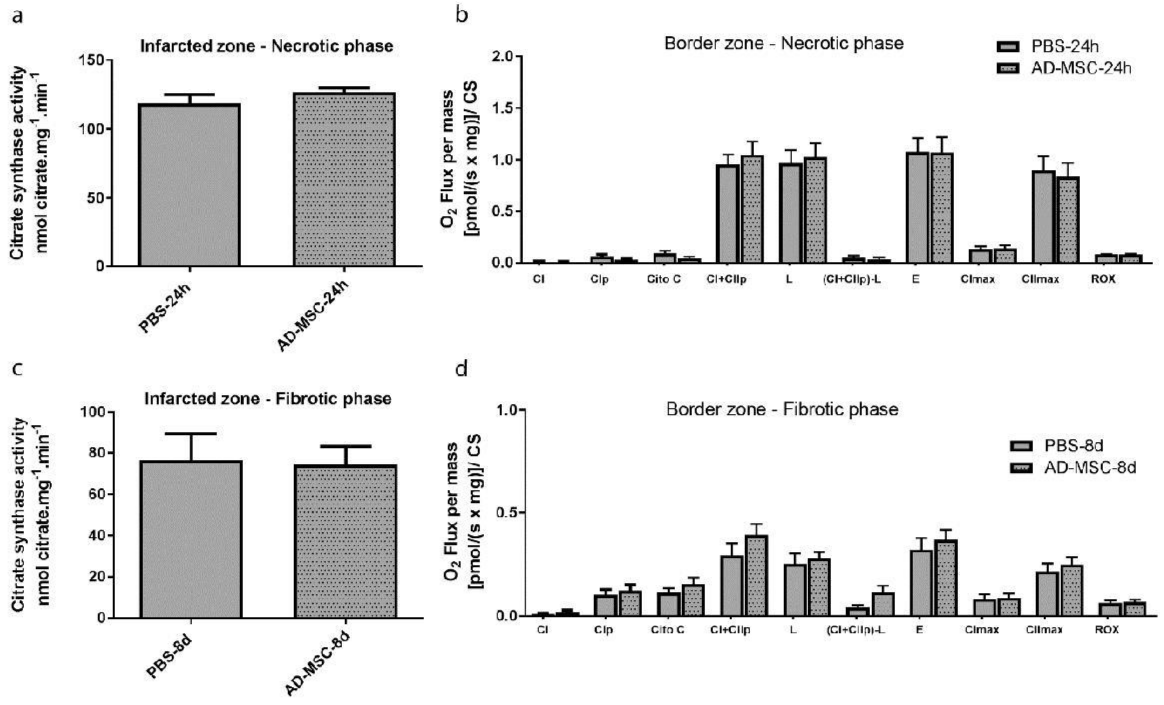

Figure.1S. Citrate synthase activity measured in cardiac muscle fibers homogenates and oxygen flux in respirometry experiments normalized to CS activity in the IZ. The activity is expressed in nmol citrate·mg^{-1}·min^{-}1 and O_2 flux by [pmol/(s·mg)]/CS. (a) CS activity comparing PBS (n=8) and AD-MSC-treatment group (n= 7) during necrotic phase and (c) fibrotic phase (PBS-8d, n=7; AD-MSC-8d group, n= 8). (b) Mitochondrial functional parameters showing O_2 flux normalized to CS activity comparing PBS-group and AD-MSC-group during necrotic phase and (d) fibrotic phase. *n* were those described in the table 1S. Data represent mean ± SEM values. To compare treated and non-treated groups was used unpaired t-test.

Table 1S. Effects of mesenchymal stromal cells from adipose tissue (AD-MSCs) on the mitochondrial function of permeabilized cardiac fibers (IZ) from rats after acute myocardial infarction (AMI). Rats were treated with AD-MSCs or PBS after AMI, and different parameters of the mitochondrial function of permeabilized cardiac fibers were analyzed by high-resolution respirometry, according to the multiple substrate-uncoupler-inhibitor titration protocol.

Mitochondrial function parameter (in pmol O_2 s^{-1} mg^{-1})	**Necrotic phase**		**Fibrotic phase**	
	**PBS (n=10)**	**AD-MSC (n=7)**	**PBS (n=7)**	**AD-MSC (n=8)**
**CI**	1.25 ± 0.56	0.91 ± 0.66	0.77± 0.41	1.44 ± 0,61
**CIp**	7.02 ± 2.18	3.73 ± 1.76	8.47 ± 2.10	8.98 ± 2.06
**CI + CIIp**	106.0 ± 10.5	131.8±15.73	23.98± 4.87	28.40± 3.71
**Leak state (L)**	107.4 ± 14.04	128.7±16.62	20.81± 3.94	20.09± 2.31
**(CI+CIIp-L)**	5.24 ± 2.5	4.13± 2.5	3.18 ± 1.17	8.3 ± 2.3
**E**	119.7 ± 14.79	134.5± 18.27	26.29± 4.68	26.66± 3.54
**CI_{max}**	14.94 ± 2.68	17.66 ± 3.8	6.78 ± 1.85	6.37 ± 1.47
**CII_{max}**	99.6 ± 15.17	104.6 ± 17.1	17.72± 3.23	18.06 ± 2.5
**ROX**	9.08 ± 0.33	10.02 ± 1.03	5.26 ± 1.02	4.9 ± 0.86
**Cytocrome C (%)**	33.16 ± 5.65	29.38 ± 6.8	20.83 ± 7.15	24.64 ± 6.7
**L/E**	0.91 ± 0.06	0.96 ± 0.04	0.79 ± 0.02	0.77 ± 0.02
**(CI+CIIp–L)/E**	0.03 ± 0,05	0.04± 0.034	0.11 ± 0.04	0.30 ± 0.09
**RCR**	1.05 ± 0.07	1.03 ± 0.03	1.14 ± 0.05	1.4 ± 0.13

CI- non-phospholylativestate;CIp-phosphorylative state associated with complex I;CI+CIIp- maximal phospholylative state; (CI+CII_p - L) - flux coupled with ATP synthesis; E - maximal respiratory capacity; CI_{max} - complex I contribution to the maximal ETS capacity; CII_{max} - complex II contribution to the maximal ETS capacity; ROX - residual oxygen consumption; Cyto C -an index of mitochondrial outer membrane integrity; L/E - leak control ratio; (CI+CIIp – L)/E- express how much from ETS capacity is used to produce ATP; RCR - respiratory control ratio. Values are expressed as mean ± SEM. Any difference was visible between vehicle-treated (PBS) group and AD-MSC-treated group (by unpaired t-test).
